# High regional variation in prostate surgery for benign prostatic hyperplasia in Switzerland

**DOI:** 10.1371/journal.pone.0254143

**Published:** 2021-07-22

**Authors:** Maria M. Wertli, Brigitta Zumbrunn, Pascal Weber, Alan G. Haynes, Radoslaw Panczak, Arnaud Chiolero, Nicolas Rodondi, Drahomir Aujesky

**Affiliations:** 1 Department of General Internal Medicine, Bern University Hospital, Inselspital, University of Bern, Bern, Switzerland; 2 CTU Bern, University of Bern, Bern, Switzerland; 3 Institute of Social and Preventive Medicine, University of Bern, Bern, Switzerland; 4 Population Health Laboratory (#PopHealthLab), University of Fribourg, Fribourg, Switzerland; 5 Department of Epidemiology, Biostatistics, Occupational Health, School of Population and Global Health, McGill University, Montreal, Canada; 6 Institute of Primary Health Care (BIHAM), University of Bern, Bern, Switzerland; University Medical Center Utrecht, NETHERLANDS

## Abstract

**Background:**

Among various treatment options for benign prostatic hyperplasia (BPH), surgical therapy is the most invasive. As Switzerland has the highest transurethral prostatectomy rate among OECD countries, we assessed the regional variation in prostate surgery for BPH and explored potential determinants of variation.

**Methods:**

We conducted a population-based analysis using discharge data for men aged ≥40 years with transurethral or simple prostatectomy from all Swiss hospitals during 2013–2018. After excluding patients with genitourinary/prostate cancer, we derived hospital service areas (HSAs) by analyzing patient flows. We calculated age-standardized mean procedure rates and variation indices (extremal quotient [EQ] and systematic component of variation [SCV]). We estimated the reduction in variance across HSAs of prostatectomy rates in multilevel regression models, with incremental adjustment for age, regional cultural and socioeconomic factors, disease burden, density of urologists, and the time since urologists’ graduation.

**Results:**

Overall, 44,253 prostatectomies (42,710 transurethral and 1543 simple) from 44 HSAs were analyzed. The mean age-standardized prostate surgery rate was 314 (range 166–500) per 100,000 men aged ≥40 years per year. The EQ was 3.01 and the SCV 5.53, indicating a high regional variation. In multivariate models, men aged 75–79 years had an 11.6-fold higher prostatectomy rate than those aged 50–54 years. French/Italian language areas had a 21% lower rate than Swiss German speaking areas. Socioeconomic factors, disease burden, and density of urologist/time since graduation were not associated with prostatectomy rates. After full adjustment, 80% of the variance in prostate surgery across HSAs remained unexplained.

**Conclusion:**

We found a remarkably high regional variation in prostate surgery rates for BPH within Switzerland.

## Background

Benign prostatic hyperplasia (BPH) increases in frequency with age and may lead to prostatic obstruction, lower urinary tract symptoms (LUTS), and urinary retention [1, 2]. Almost 50% of men have at least moderate lower urinary tract symptoms in the eighth decade of life [2]. Transurethral resection of the prostate (TURP) and open simple prostatectomy were historically the standard treatments to remove prostatic obstruction [3]. These procedures require however hospitalization and carry a risk of bleeding, sexual dysfunction, urinary incontinence, and urethral stricture/bladder neck contracture [4, 5]. Over the last 30 years, a multitude of treatments for BPH, including drugs (α-adrenergic antagonists, 5-α reductase inhibitors) and minimally invasive surgical techniques with the potential to reduce the need for hospitalization and complications [3, 6], were introduced into clinical practice [7]. Guidelines recommend conservative measures, including behavioral changes and drugs, as the first-line treatment for men with BPH who are bothered by their LUTS [8–10]. Surgery is recommended when patients have BPH-related complications (e.g., urinary retention, renal insufficiency, or recurrent urinary tract infections), or when patients fail to improve with conservative therapy or refuse drug treatment. However, only few studies directly compared the effectiveness, safety, patient satisfaction, and costs of drug vs. surgical treatment for symptomatic BPH and the optimal timing to switch from conservative to surgical treatment is not well defined [11, 12]. Limited evidence suggests that surgery may be more cost-effective in the long run and in patients with severe LUTS [13, 14]. In the USA and other countries, a decrease in surgery for BPH has been observed over time [15, 16].

**Fig 1:**
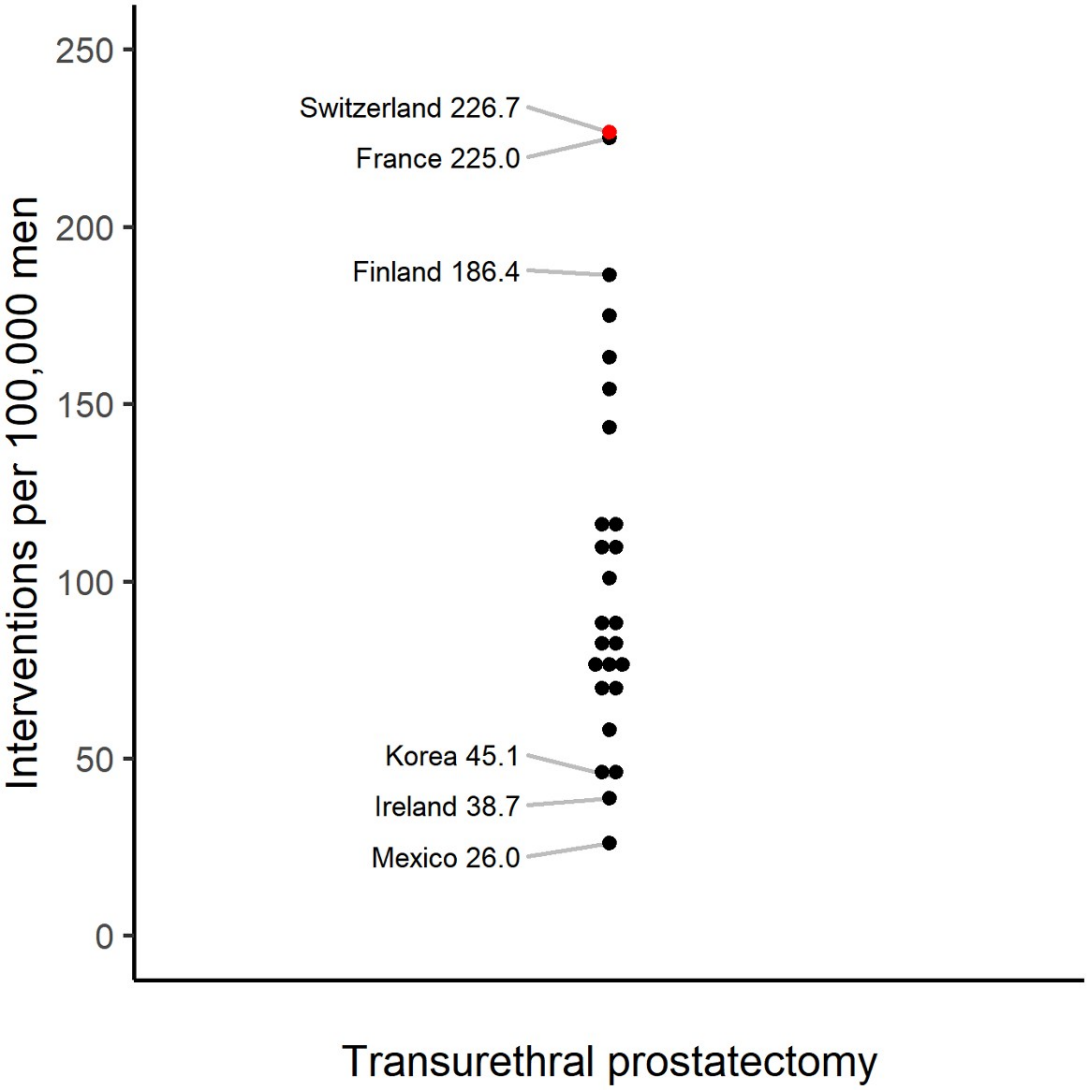
Comparison of transurethral prostatectomy rates across OECD countries (incl. Costa Rica) in 2018. Each dot represents the crude transurethral prostatectomy rate per 100,000 men in a given country. The red dot indicates Switzerland.

Although transurethral prostatectomy rates decreased by 11% in Switzerland between 2008 and 2018, Switzerland had the highest procedure rates among Organization for Economic Co-operation and Development (OECD) countries during this period, with 227 interventions per 100,000 men in 2018 (Fig 1) [17]. However, whether prostatectomy rates are uniformly high across Swiss regions and which factors drive prostatectomy rates in Switzerland is largely unknown. The aim of this study was therefore to assess regional variations in prostatectomy rates using Swiss national data and to determine whether demographic, cultural, socioeconomic, health, and supply factors explain such variation.

## Methods

### Data sources

We conducted a population-based, small area variation analysis using routinely collected patient discharge data from all Swiss public and private acute care hospitals and census data for calendar years 2013–2018. The method has been described previously [18, 19]. Swiss hospitals are legally obligated to provide the Swiss Federal Statistical Office (SFSO) with an anonymized, standardized data set for each hospital discharge, which includes demographic information, procedure codes based on the Swiss Classification of Operations (CHOP; an adaptation of the U.S. ICD-9-CM volume 3 procedure classification), and diagnostic codes based on the International Classification of Diseases, 10^th^ revision, German Modification (ICD-10-GM) [20]. Further, the area of patient residence and hospital location within one of 705 Swiss MedStat regions (administrative regions based on aggregated ZIP-codes) are recorded [21]. The Swiss Hospital Discharge Masterfile covers 100% of discharges and the SFSO reviews data integrity and completeness [22].

We used Swiss National Cohort data from 2014 to determine the main language (German, French, or Italian) [23] and data from the SFSO to determine the population density for each MedStat region. We abstracted measures of socioeconomic status (neighborhood information on rent, education, occupation, and crowding) using Swiss census data from 2012 to 2015 [24]. Finally, we obtained the number of urologists per MedStat region and their time since graduation from medical school for 2014 from the Swiss Medical Association. Because our study was based on anonymized administrative data only, it was exempted from ethics committee approval according to the Swiss Human Research Act.

### Derivation of Swiss Hospital Service Areas

Switzerland has compulsory basic health insurance coverage, with voluntary semiprivate and private insurance plans covering additional medical services. Although Swiss hospital care is primarily organized based on 26 geographic regions (cantons), patients may utilize hospital services outside their canton of residence and the use of cantons as a unit of observation may skew procedure rates. We therefore used a fully automated method to generate reproducible general hospital service areas (HSAs) using all patient discharge data from the calendar years 2013–2016 (data that was available when the general HSAs were derived) [18]. Briefly, we identified 4,105,885 patient discharges aged ≥18 years from 155 Swiss public and private acute care hospitals during calendar years 2013–2016. Only patients living in Switzerland were considered. We analyzed the flows and assigned MedStat regions from which the highest proportion of residents was discharged to the same HSA (plurality rule). HSAs with less than 50% of the patients being treated within the same HSA or less than 10 discharges were merged with the neighboring HSA which received most discharges, yielding 63 general HSAs. We then identified patient discharges with specific CHOP codes for transurethral (CHOP 60.20–22, 60.29, 60.61.10, 60.95–97, 60.99.2) and simple supra-/or retropubic prostatectomy (CHOP 60.3, 60.4) from all Swiss acute care hospitals from 2013–2018. As prostatectomies are procedures that are not performed in every hospital, we further collapsed the general HSAs into intervention-specific HSAs by aggregating the 63 general HSAs into 44 intervention-specific HSAs, using the method described above. We then drew choropleth maps of the 44 final HSAs using Geographical Information System (GIS)-compatible vector files.

### Study population

Overall, we identified 57,976 discharges with specific codes for transurethral and simple prostatectomy. We excluded those with genitourinary cancer, including prostate cancer, (n = 13,625), genitourinary injuries (n = 32), congenital disease of the prostate (n = 2), and men under 40 (n = 64), leaving a final study sample of 44,253 patient discharges for prostate surgery. Of these, 42,710 underwent a transurethral and 1543 a simple prostatectomy. Those who underwent both types of procedures were assigned to the more invasive simple prostatectomy.

### Measures of variation

We calculated age-standardized prostatectomy rates per 100,000 men aged ≥40 years for each HSA using procedure counts and 2013–2018 census data for the adult Swiss population [25]. We used direct standardization with age bands 40 to 44, 45 to 49, 50 to 54, …,75 to 79, 80+. To examine the variation in prostatectomy rates across Swiss HSAs, we determined the extremal quotient (EQ), which is the highest divided by the lowest procedure rate. While the EQ is an intuitive measure of variation, it is prone to distortion by extreme values [26]. We also calculated the coefficient of variation (CV), i.e., the ratio of the standard deviation of the procedure rates to the mean rate, and the systematic component of variation (SCV) [26, 27]. Although less intuitive than the EQ, the SCV represents the non-random part of the variation in procedure rates while reducing the effect of extreme values [26, 27]. The SCV is derived from a model that recognizes the differences in rates across areas and the random variation within each area’s true rate. An SCV of 5.4–10 is considered indicative of high variation and an SCV of >10 of very high variation [26, 28].

### Determinants of variation

Differences in illness incidences and socioeconomic factors are possible and legitimate causes of variation [26]. We therefore explored four regional domains that could influence prostate surgery rates: demographics (age), cultural (language region) and socioeconomic factors (median density of male population, Swiss neighborhood index of socioeconomic position [SSEP], insurance status, and Swiss citizenship), disease burden of the male population, and supply factors (density of urologists per 10,000 men and their average time since graduation). The language spoken by the majority of people living in an HSA was used to classify each HSA as either Swiss German or French/Italian language region as a proxy for culture. We used population density as a proxy for the level of urbanization a resident lives in. The socioeconomic status of each HSA was measured using the mean SSEP of residents of a given HSA [24]. The SSEP consists of four domains (median rent per m^2^, proportion of households led by a person with no/low education, proportion headed by a person in manual/unskilled occupation, and mean crowding, all on the neighborhood level). The SSEP varies between 0 (lowest) and 100 (highest) and correlates well with mortality [29]. The percentage of discharges with semiprivate/private vs. general health insurance and Swiss vs. foreign citizenship was used as an additional measure of the socioeconomic status of each HSA. As a proxy for the population burden of disease, we calculated age-standardized incidence rates of hip fractures, cancers of the colon or lung treated surgically, acute myocardial infarctions, or strokes for men in each HSA, as differences in these disease rates are likely to reflect true regional differences in burden of disease rather than differences in coding intensity or supply factors [30, 31]. The density of urologists and their average time since graduation were used as proxies for health services availability and physician training, respectively.

To explore determinants of prostate surgery rates in Switzerland, we used progressively adjusted multilevel Poisson regression with a log link to model the procedure rates in each HSA. Age was used in the bands 40 to 44, 45 to 49, …, 75 to 79, 80+ and adjustment was performed on an HSA level. Model 1 included only the calendar year of the procedure. Model 2 was additionally adjusted for demographics (age). Model 3 was additionally adjusted for HSA-level language and socioeconomic factors (male population density, SSEP, insurance status, and Swiss citizenship). Model 4 was further adjusted for HSA-level male population burden of disease. Model 5 was additionally adjusted for the density of urologists per HSA and their average time since graduation. HSA was included as a random intercept in all models. All covariates were selected *a priori*. We depicted the variation in HSA rates as average predicted prostate surgery rates per 100,000 men aged ≥40 years per HSA derived from the multilevel regression models. Where rates are shown in maps, categories for the rates were chosen to be about equal in width.

We expressed the impact of determinants on prostatectomy rates as incidence rate ratios (IRRs), defined as the prostatectomy rate in the defined category (e.g., French/Italian language region) relative to the estimated prostate surgery rate in the reference category (e.g., German language region). We also determined the percentage reduction in procedure variation across the 44 HSAs by examining the variance of the random intercept. We considered the residual, unexplained variation of the fully adjusted model a proxy for unwarranted variation, i.e. the variation that cannot be attributed to potential patient need [26, 32–34]. Statistical analyses were performed using Stata version 16.1 (StataCorp, College Station, TX, USA) and R statistical software version 3.6.1 [35].

## Results

### Characteristics of Swiss HSAs and the study population

Of 44 HSAs, 29 were located in the Swiss German and 15 in the French/Italian-speaking part of Switzerland. The median male adult population per HSA was 39,543 persons (interquartile range [IQR] 22,430–91,959), with a median population density of 125 men per km^2^ (IQR 48–203), and a mean SSEP of 54 points (IQR 51–59). The average proportion of residents with semi-private/private insurance and Swiss nationality per HSA was 21% (IQR 17–28) and 82% (IQR 78–86), respectively. The median burden of disease was 0.59 (IQR 0.53–0.65) comorbidities per 1000 men. The median density of urologists was 8.2 (IQR 6.0–12.0) per 10,000 men (S1 Fig), with a median average time since graduation of 23 years (IQR 20–26).

The majority of the 44,253 patients discharged after a prostatectomy were 65 years or older (72%), had general health insurance (65%), and were Swiss nationals (88%) (Table 1). Almost all patients had undergone transurethral prostate surgery (97%).

**Table 1:** Characteristics of 44,253 men aged ≥40 years with prostate surgery from 2013 to 2018.

Characteristic	n (%)
Age, years	
40–49	607 (1)
50–59	5409 (12)
60–69	14,651 (33)
70–79	16,497 (37)
80+	7089 (16)
Insurance class	
General	28,891 (65)
(Semi-)private	15,362 (35)
Citizenship	
Swiss	39,142 (88)
Non-Swiss	5111 (12)
Procedure type	
Transurethral prostatectomy	42,710 (97)
Simple supra- or retropubic prostatectomy	1543 (3)

### Variation in procedure rates across Swiss HSAs

**Fig 2:**
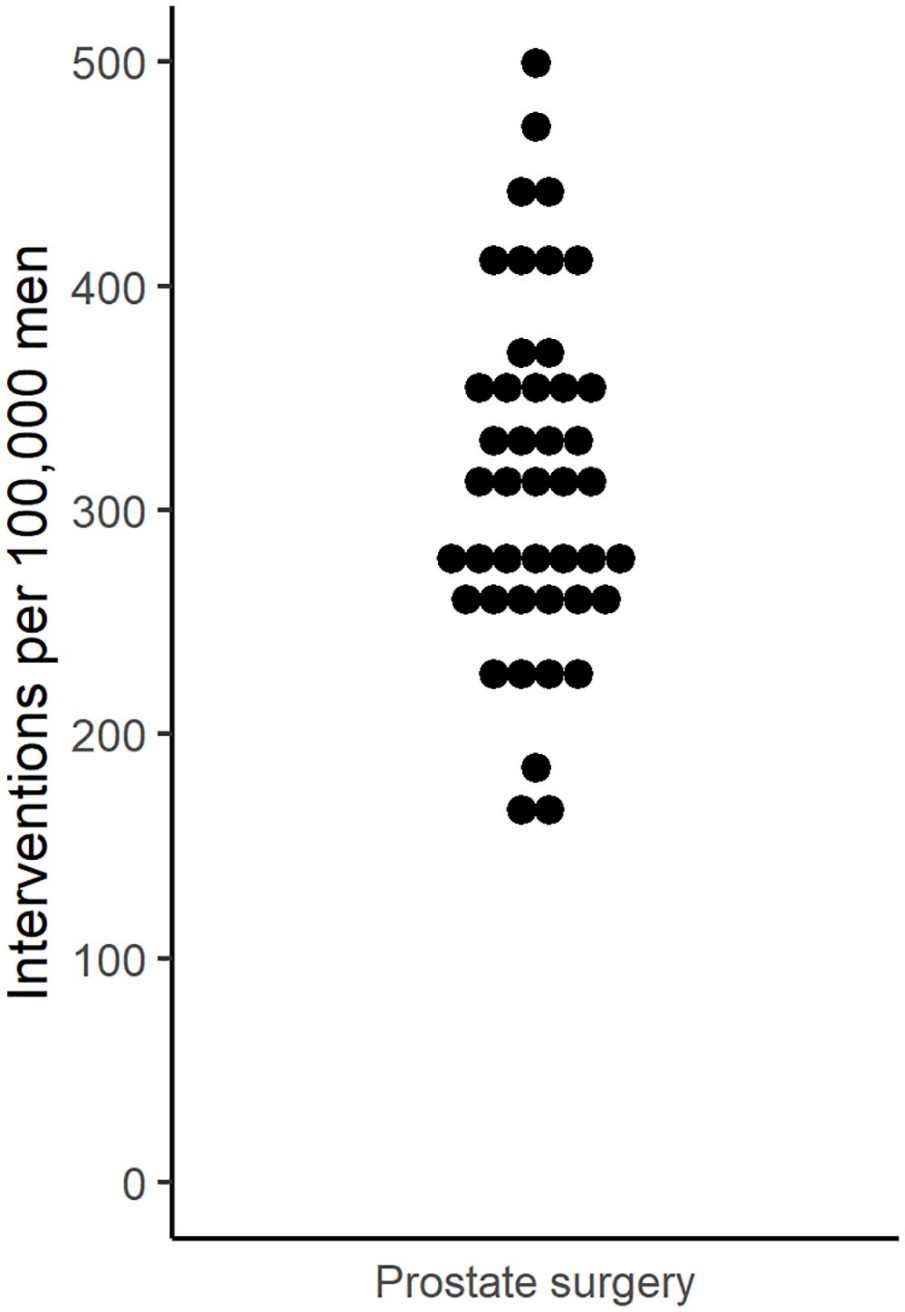
Variation in age-standardized prostate surgery rates across 44 Swiss Hospital Service Areas. Each dot represents a Hospital Service Area.

The mean age-standardized prostate surgery rate was 314 (range 166–500) per 100,000 men ≥40 years of age per year (Fig 2). Detailed age-standardized prostate surgery rates for each HSA are shown in the S1 Table. The EQ was 3.01, the CV 0.25, and the SCV 5.53, indicating a high variation (Table 2). We could discern no clear time trends in the variation among HSAs between 2013 and 2018.

**Table 2:** Variation in prostate surgery rates across 44 Swiss Hospital Service Areas.

	Overall	2013	2014	2015	2016	2017	2018
EQ	3.01	3.81	3.46	3.57	4.38	3.05	3.61
CV	0.25	0.28	0.34	0.30	0.28	0.24	0.29
SCV	5.53	5.87	8.72	6.62	6.04	4.09	6.29

Abbreviations: EQ, extremal quotient; CV, coefficient of variation; SCV, systematic component of variation.

**Fig 3:**
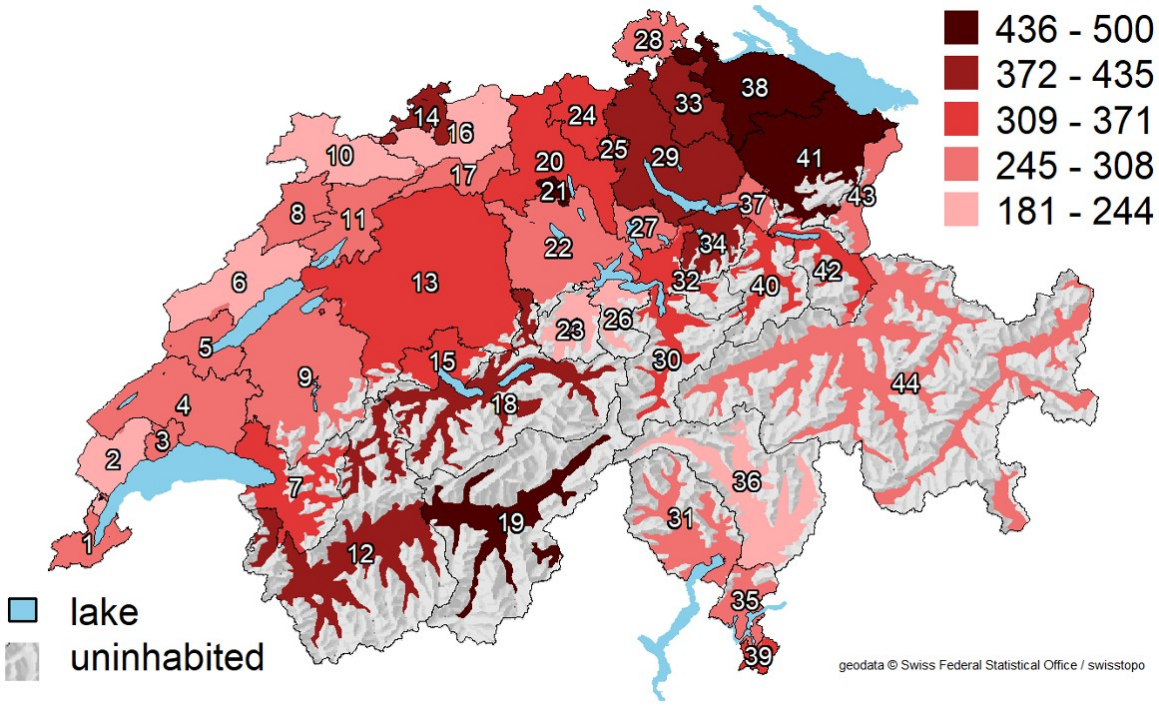
Map of the average adjusted predictions of prostate surgery rates across 44 Swiss Hospital Service Areas. Average predicted prostate surgery rates for each HSA are shown as red-scale categories per 100,000 men ≥40 years of age. Adjusted for procedure year, age, language region, density of male population, mean Swiss Neighborhood Index of Socioeconomic Position, insurance status, Swiss citizenship, disease burden of the male population, density of urologists, and urologists’ time since graduation. Rate categories were chosen to be about equal in width. Shaded relief map reprinted from the Federal Office of Topography swisstopo, Switzerland https://shop.swisstopo.admin.ch/en/products/maps/overview/relief and shape files derived from postcode-level shape file used to create map of Switzerland, e.g., https://www.geocat.admin.ch/) under a CC BY license, with permission from Alexandra Frank, original copyright 2006.

After full adjustment for procedure year, age, language region, socioeconomic factors, burden of disease, and urologist density/time since graduation, the average predicted prostate surgery rates per HSA varied between 182 and 500 per 100,000 men ≥40 years of age (Fig 3), of which four were above 436 per 100,000 (HSA number 19, 21, 38, and 41, located in different parts of Switzerland) and seven were below 244 per 100,000 (HSA number 2, 6, 10, 16, 23, 26, 36, also spread across the country). After full adjustment, 80% of the variance in prostate surgery rates amongst HSAs remained unexplained.

### Determinants of variation in procedure rates

Procedure rates decreased by 3% per year (IRR 0.97; 95% CI 0.96–0.97; Table 3). Age was the most powerful predictor of procedure variation across HSAs. Compared to men aged 50–54 years, men aged 75–79 years had an 11.6-fold higher prostate surgery rate (IRR 11.57; 95% CI 10.97–12.21). French/Italian language areas showed a 21% lower prostatectomy rate (IRR 0.79; 95% CI 0.66–0.94) than Swiss German-speaking areas. None of the other predictors were statistically significantly associated with prostatectomy rates.

**Table 3:** Determinants of variance in the incidence rates of prostate surgery across 44 Swiss Hospital Service Areas.

Determinants		Model 1*	Model 2^†^	Model 3^†^	Model 4^#^	Model 5^&^
		Incidence rate ratio (95% confidence interval)§				
Procedure year (per year)		0.99 (0.98–0.99)	0.97 (0.97–0.98)	0.97 (0.96–0.98)	0.97 (0.96–0.97)	0.97 (0.96–0.97)
Age category (years)	40–44		0.07 (0.06–0.09)	0.07 (0.06–0.09)	0.07 (0.06–0.09)	0.07 (0.06–0.09)
	45–49		0.31 (0.28–0.34)	0.31 (0.28–0.34)	0.31 (0.28–0.34)	0.31 (0.28–0.34)
	50–54		Reference	Reference	Reference	Reference
	55–59		2.67 (2.52–2.83)	2.67 (2.52–2.83)	2.67 (2.52–2.83)	2.67 (2.52–2.83)
	60–64		5.28 (5.00–5.57)	5.28 (5.00–5.57)	5.28 (5.00–5.57)	5.28 (5.00–5.57)
	65–69		8.28 (7.86–8.73)	8.28 (7.86–8.73)	8.28 (7.86–8.73)	8.28 (7.86–8.73)
	70–74		10.75 (10.20–11.33)	10.75 (10.20–11.33)	10.75 (10.20–11.33)	10.75 (10.20–11.33)
	75–79		11.57 (10.97–12.21)	11.57 (10.97–12.21)	11.57 (10.97–12.21)	11.57 (10.97–12.21)
	80+		9.26 (8.77–9.77)	9.26 (8.77–9.77)	9.26 (8.77–9.77)	9.26 (8.77–9.77)
Language region	Swiss German			Reference	Reference	Reference
	French/Italian			0.79 (0.68–0.93)	0.79 (0.67–0.93)	0.79 (0.66–0.94)
Density of male population (per 100/km^2^)				1.02 (0.95–1.09)	1.02 (0.96–1.09)	1.02 (0.96–1.10)
Mean SSEP (per 1 unit increase)				1.01 (0.99–1.03)	1.01 (1.00–1.03)	1.01 (0.99–1.03)
(Semi-)private insurance (per 10% increase)				0.88 (0.78–1.00)	0.88 (0.78–1.01)	0.89 (0.78–1.01)
Swiss citizenship (per 10% increase)				0.93 (0.85–1.01)	0.92 (0.84–1.01)	0.93 (0.85–1.01)
Burden of disease (per comorbidity/1000 men)т					1.06 (0.97–1.16)	1.06 (0.97–1.16)
Density of urologists (per urologist/10,000 men)**						1.00 (0.98–1.01)
Average time since graduation (per 5 years)						1.00 (0.93–1.07)
**Remaining variance from Model 1 (%)**^II^			**102.1**	**79.9**	**80.9**	**80.0**

Abbreviations: SSEP, Swiss Neighborhood Index of socioeconomic position.

*Model 1: adjusted for procedure year.

^†^Model 2: additional adjustment for age.

^‡^Model 3: additional adjustment for cultural and socioeconomic factors (language region, density of men, SSEP, insurance status, and Swiss citizenship).

^#^Model 4: additional adjustment for population burden of disease.

^&^Model 5: additional adjustment for density of urologists and their average time since urologists’ graduation.

^§^Prostate surgery rate in the defined category relative to the reference category. For instance, the incidence rate ratio of 0.79 indicates a 21% lower prostate surgery rate in French/Italian-speaking than in Swiss German speaking language areas.

^т^Burden of disease is defined as the sum of age-standardized incidence rates for the following comorbidities: hip fracture, colon or lung cancer treated surgically, acute myocardial infarction, and stroke. The incidence rate ratio is the increase (decrease) in procedure rates when the regional burden of disease increases by 1 comorbidity per 1000 men.

**The incidence rate ratio is the increase (decrease) in procedure rates when the regional density of urologists increases by 1 urologist per 10,000 men.

^II^Expresses the variance around the mean prostate surgery rate.

## Discussion

Our study demonstrates a rather high rate of prostate surgery for BPH in Switzerland and a high variation in prostate surgery among Swiss regions. This variation remained largely unexplained by procedure year, demographic, cultural and socioeconomic factors, disease burden, and the density of urologists.

Transurethral prostatectomy rates and time trends differ greatly across OECD countries [17]. These between-country differences may reflect differences in a multitude of factors, including demographic composition, patient preferences, socioeconomic deprivation with limited access to care, physicians’ clinical decisions, health service configuration and financing, and the availability of specialists [26]. Switzerland has one of the highest health care expenditure per capita across OECD countries [36], a number of doctors per capita above the OECD average, and a high specialist-generalist ratio. Swiss residents also benefit from universal health care coverage and have timely access to care. These factors could contribute to the high prostatectomy rate in Switzerland compared to other countries.

The remarkably high variation of prostate surgery for BPH across geographically closely related Swiss regions, largely (>80%) was not associated with demographic, cultural, socioeconomic, health, and supply factors, is more difficult to interpret. As it is implausible that men from neighboring areas differ in terms of size/anatomy of their prostate or their preferences for outcomes associated with prostatectomy, the variation may be unwarranted and explained by Swiss urologists’ differing practice styles.

In the 1980 and 1990s, Wennberg and colleagues found a similarly high variation in overall prostatectomy rates for various indications within different health care systems (SCV 5.0–9.3) [27] and TURP for BPH in the USA (SCV 5.2) [37]. They identified two clinical sources of unwarranted variation in prostatectomy for BPH [38]: (1) lack of information concerning the risks/benefits of the procedure, and (2) failure to base decisions on patient preferences for outcomes, as patients with similar LUTS report considerable difference in the degree to which they are bothered [39].

Several decades later, the high regional variation in prostatectomy for BPH persists in Switzerland and other countries [40, 41]. Over the last three decades, there was a proliferation of pharmacological and minimally invasive surgical treatments for BPH [3]. Although the European Urology Association and the American Urological Association publish regularly updated BPH guidelines since the early 2000s [42, 43], this apparently has not led to consolidation of practice into a single best practice [12]. Both guidelines recommend conservative management as the initial treatment for uncomplicated BPH and underline the importance of discussing the risks and benefits of various treatment options. However, guideline adherence is highly variable among urologists and may be due to the limited evidence on best evaluative practices and a lack of comparative effectiveness studies to help guide the multiple existing treatment choices and the optimal timing for prostate surgery in BPH [11, 12, 44–47]. The same can be said about primary care physicians who are highly variable in their referral thresholds to urologists [48]. According to an international survey, urologists have discrepant attitudes to counselling patients on side effects related to BPH/LUTS treatments, and most urologists do not discuss alternative treatments with patients based on the risk of different outcomes [49].

Evidence suggests that adherence to guideline-recommended evaluative care work-up for BPH translates into lower prostate surgery rates. In an analysis of Medicare beneficiaries with BPH, a high guideline compliance was associated with a 91% decrease in the adjusted odds of receiving prostate surgery [50]. Shared decision-making programs/decision aids for symptomatic BPH result in increased patient knowledge about their condition and a higher satisfaction with treatment choice but do not necessarily result in lower rates of prostate surgery [51–54]. While there is no published evidence about differing guideline adherence and shared decision-making processes among Swiss urologists, such differences could at least partially be responsible for the observed regional procedure variation in our study.

As the prevalence of BPH increases with age [1, 2], it is not surprising that age was the most important predictor of prostate surgery in our analysis. French/Italian speaking areas had a 21% lower prostate surgery rate than Swiss German language regions, possibly due to more conservative physician practice styles or patient preferences for less invasive treatments. We previously have observed lower rates of other preference-sensitive surgical interventions in the French/Italian speaking parts of Switzerland, including vertebroplasty, hysterectomy and joint replacement [19, 55, 56]. We found no association between semi-/private insurance and prostatectomy rates, arguing against the suspicion that a semi-/private insurance (which results in higher physician fees) may fuel overtreatment in Switzerland. Similarly, we found no association between the density of urologists and procedure rates. Several high-volume areas (e.g., HSA 11, 18) had a relatively low density of urologists, indicating that procedure variation may be due to local urologists’ attitudes and not their number.

Our study has several potential limitations. First, we did not have clinical data and could not verify the exact indication for prostate surgery and whether it was adherent to guideline recommendations for treating BPH. Second, we had no information about regionally differing physician or patient attitudes towards prostate surgery for BPH. Third, our analysis was limited to inpatient procedures and did not include prostate surgery done in the outpatient setting. However, this is not a major limitation, as >98% of transurethral prostatectomies in Switzerland are done on an inpatient basis [17]. Fourth, Swiss coding practices did not allow a regional comparison of different surgical techniques over time, including the uptake of minimally invasive methods, such as water vapor thermal therapy, prostatic urethral lift, and robot-assisted and laparoscopic simple prostatectomy [3]. Fifth, adjustment for ecological variables on a population level (i.e., age, language, SSEP, insurance, citizenship, and burden of disease) includes a risk of ecological fallacy by drawing conclusions about the behavior of individuals based on population level parameters [57]. Finally, our results describe associations and cannot infer causality.

In conclusion, we found a remarkably high regional variation in prostate surgery rates for BPH within Switzerland. The larger part of the variation was not associated with procedure year, age, cultural and socioeconomic factors, disease burden, and the density of urologists/time since graduation.

## Supporting information

S1 Fig Density of urologists (number per 10,000 men) across 44 Swiss Hospital Service Areas. Shaded relief map reprinted from the Federal Office of Topography swisstopo, Switzerland https://shop.swisstopo.admin.ch/en/products/maps/overview/relief and shape files derived from postcode-level shape file used to create map of Switzerland, e.g., https://www.geocat.admin.ch/) under a CC BY license, with permission from Alexandra Frank, original copyright 2006.
(TIF)

S1 Table Age-standardized prostate surgery rates per year for 44 Hospital Service Areas. Abbreviation: HSA, Hospital Service Area.
(DOCX)
