# Supplementary figures and images for: High regional variation in prostate surgery for benign prostatic hyperplasia in Switzerland
Source: PLoS One. 2021 Jul 22;16(7):e0254143. doi: 10.1371/journal.pone.0254143 (PMC8297757; doi:10.1371/journal.pone.0254143)

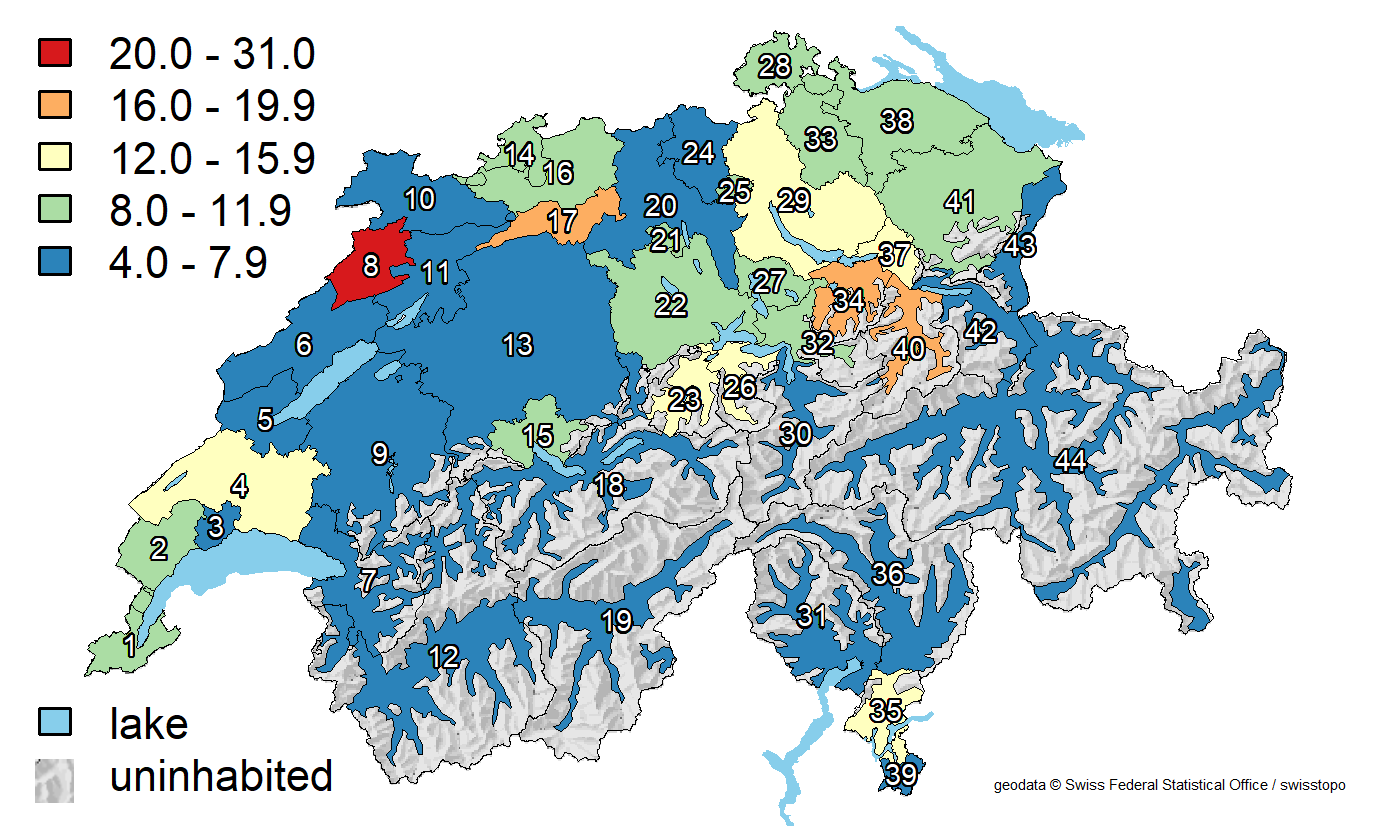

Supplement: S1 Fig — Shaded relief map reprinted from the Federal Office of Topography swisstopo, Switzerland https://shop.swisstopo.admin.ch/en/products/maps/overview/relief and shape files derived from postcode-level shape file used to create map of Switzerland, e.g., https://www.geocat.admin.ch/) under a CC BY license, with permission from Alexandra Frank, original copyright 2006. (TIF) [file pone.0254143.s001.tif]
