# Supplementary material for: High regional variation in prostate surgery for benign prostatic hyperplasia in Switzerland
Source: PLoS One. 2021 Jul 22;16(7):e0254143. doi: 10.1371/journal.pone.0254143 (PMC8297757; doi:10.1371/journal.pone.0254143)
Supplement: S1 Table — Abbreviation: HSA, Hospital Service Area. (DOCX) [file pone.0254143.s002.docx]

**S1 Table**

| **HSA number** | **Interventions per 100,000 men aged ≥40 years** |
| --- | --- |
| 36 | 166 |
| 23 | 167 |
| 10 | 185 |
| 26 | 220 |
| 16 | 231 |
| 31 | 232 |
| 6 | 234 |
| 8 | 254 |
| 11 | 255 |
| 22 | 255 |
| 17 | 255 |
| 35 | 260 |
| 28 | 267 |
| 44 | 271 |
| 2 | 272 |
| 5 | 276 |
| 4 | 281 |
| 37 | 281 |
| 3 | 283 |
| 27 | 286 |
| 43 | 306 |
| 9 | 308 |
| 1 | 308 |
| 39 | 310 |
| 15 | 319 |
| 40 | 324 |
| 42 | 330 |
| 30 | 333 |
| 13 | 338 |
| 18 | 347 |
| 25 | 347 |
| 24 | 353 |
| 7 | 361 |
| 20 | 363 |
| 32 | 364 |
| 14 | 376 |
| 34 | 408 |
| 12 | 408 |
| 29 | 414 |
| 33 | 415 |
| 41 | 441 |
| 38 | 443 |
| 19 | 471 |
| 21 | 500 |
